# An Analysis of High-Resolution Computed Tomography Chest Manifestations of COVID-19 Patients in Pakistan

**DOI:** 10.7759/cureus.9373

**Published:** 2020-07-24

**Authors:** Maria Khaliq, Riffat Raja, Nasir Khan, Hina Hanif

**Affiliations:** 1 Radiology, Holy Family Hospital/Rawalpindi Medical University, Rawalpindi, PAK; 2 Radiology, Rawalpindi Medical University, Rawalpindi, PAK

**Keywords:** covid-19, hrct chest, ct-ss, ground glass opacities, crazy paving

## Abstract

Objective

The objective of the present study is to describe high-resolution CT (HRCT) chest manifestations of coronavirus disease 2019 (COVID-19) patients presenting to a tertiary healthcare facility in Punjab, Pakistan, and to analyze the distribution of the disease in lung fields. Additionally, we assess the role of chest CT severity scoring (CT-SS) in determining the severity of pneumonia.

Methods

In this cross-sectional descriptive study conducted from March 30, 2020, to May 30, 2020, 87 confirmed COVID-19 patients undergoing HRCT scan in a tertiary care facility in Punjab, Pakistan were included. The HRCT chest was performed on the patients using a standard protocol. Each study was evaluated for the presence of ground-glass opacities (GGOs), consolidation, mixed pattern, distribution, crazy paving, reverse halo sign, nodules, pleural effusion, and other findings. Additionally, CT-SS was calculated by dividing each lung into 20 zones. Each zone was scored as 0, 1, and 2, representing no involvement, <50% involvement, and >50% involvement of one zone respectively (total score: 0-40 for each patient). The patients were classified into mild, moderate, and severe cases (mild: CT-SS of <20, moderate: CT-SS of 20-30, and severe: CT-SS of >30).

Results

GGO was the most common finding, as seen in 88.5% of the patients, followed by consolidations (52.8%) and crazy paving (33.3%). The majority of the patients showed the bilateral and peripheral distribution of the disease process. Vascular dilatation and bronchiectasis were seen in 10 patients; pleural effusions were observed in only two study patients, while no patient exhibited reverse halo sign or pulmonary nodules. The superior segment of lower lobes was the most commonly involved segment bilaterally. According to CT-SS, 78 (89.6%), six (6.9%), and three (3.45%) patients had mild, moderate, and severe disease respectively.

Conclusion

The typical imaging findings of COVID-19 on HRCT are GGOs with multilobe involvement and bilateral, peripheral, and basal predominance. CT-SS is helpful in categorizing pneumonia into mild, moderate, and severe types, thereby helping to identify patients with severe disease. This is particularly helpful in settings where fast triage is required.

## Introduction

The global pandemic of coronavirus disease 2019 (COVID-19) has been progressing rapidly and worsening around the world, and specialists in a wide variety of medical and diagnostic fields have been contributing their expertise regarding early diagnosis and management of the disease. Reverse transcriptase-polymerase chain reaction (RT-PCR) test is considered the gold standard for diagnosing COVID-19 [1]. However, there are certain limitations to this test. It is time-consuming, is not readily available everywhere, and can also give false-negative results. Moreover, studies have shown that it has a low sensitivity of 60-71% [2,3,4]. This low sensitivity is mainly attributed to low viral load and laboratory errors [5]. Because of the high number of false-negative results, repeat testing is often required. This is particularly challenging due to issues related to infrastructure and the availability of test kits. Recent studies have shown that CT can be employed as a complement to RT-PCR for diagnosing COVID-19 [5,6]. High-resolution CT (HRCT) of the chest is now commonly used not only in the diagnosis but also in monitoring the progression of COVID-19 pneumonia. HRCT chest has a sensitivity of 56-98% in the diagnosis of COVID-19 pneumonia [3,4]. However, experts still argue over the utility of CT as a diagnostic test for COVID-19 pneumonia, and the role of CT in patients with COVID-19 pneumonia is poorly understood.

Typical imaging features of COVID-19 on CT chest include ground-glass opacities (GGOs) and consolidation with bilateral and multilobe involvement and basal and peripheral predominance [5]. However, most of the available literature on this topic is reported and published from Far East [5,7,8]. Lung changes seen on CT chest are similar to those encountered in other viral types of pneumonia. Therefore, it is argued that although CT chest is a sensitive modality to diagnose COVID-19 pneumonia, it lacks the specificity. In this study, we describe the typical imaging features of COVID-19 pneumonia on CT chest in patients presenting to a tertiary care hospital in Punjab, Pakistan, and analyze the distribution of the disease in lung fields. We also assess the role of chest CT severity scoring (CT-SS) in determining the severity of pneumonia. We believe our findings will help in the identification and management of patients with severe disease, especially in settings where fast triage is required.

## Materials and methods

Study design

We opted for a cross-sectional descriptive study method.

Inclusion criteria

All patients with confirmed COVID-19 infection who underwent an HRCT scan of the chest at a tertiary healthcare facility in Punjab, Pakistan from March 30, 2020, to May 30, 2020, were included in the study.

Exclusion criteria

Patients who were not willing to be a part of the study were excluded.

Data collection and analysis

After obtaining informed written consent, HRCT chest scans were performed on the patients using the standard protocol. Scans were performed with a multidetector (16) Toshiba Aquilion CT scanner (Canon Medical Systems Corporation, Ōtawara, Japan) with tube voltage and current set at 120 kvp and 350 mA respectively. Each patient was scanned in a supine position, and lung fields from apices to bases were scanned in single breath-hold and 1-mm slice thickness. The images were analyzed at the workstation by two radiologists separately. Each study was evaluated for the presence of ground-glass haze/opacities, consolidation or mixed pattern, distribution of opacities, i.e., central/peripheral/mixed pattern, crazy paving, reverse halo sign, nodules, pleural effusion, and other findings. GGO is defined as an area of increased attenuation where the underlying vessels are not obscured. Consolidation is defined as an area of increased attenuation in which underlying vessels are obscured. The crazy paving pattern is defined as a geographical area of ground-glass haze with inter and intralobular septal thickening. The outer one-third of the lung was taken as peripheral while the remaining part of the lung was considered as central.

Additionally, the CT-SS was assessed for each patient. Both lungs were divided into 20 zones. Each anatomical lung segment was considered as a single zone except in the left upper lobe where the apicoposterior segment was considered as apical and posterior, i.e., two separate zones. Similarly, the posteromedial basal segment of the left lower lobe was considered as two separate zones, i.e., posterior basal and medial basal zones. The involvement of each zone was scored as 0, 1, and 2. A score of 0 indicated no involvement, a score of 1 indicated <50% involvement of the zone and a score of 2 indicated >50% involvement of one zone. The total score ranged from 0-40 for each patient. According to the scores, the patients were classified into mild, moderate, and severe cases. Mild disease indicated a score of <20. Moderate cases had scores of 20-30 and severe cases showed scores of >30.

Data was entered and analyzed using SPSS Statistics version 23.0 (IBM, Armonk, NY) for manifestations and their percentages with reference to the parameters described above. The data was then compared with the available literature on this subject.

## Results

A total of 87 patients who underwent HRCT of the chest fulfilled the inclusion criteria. Among those, 45 (51.7%) were males and 42 (48.2%) were females. The average age of the study patients was 47.24 years.

**Figure 1:**
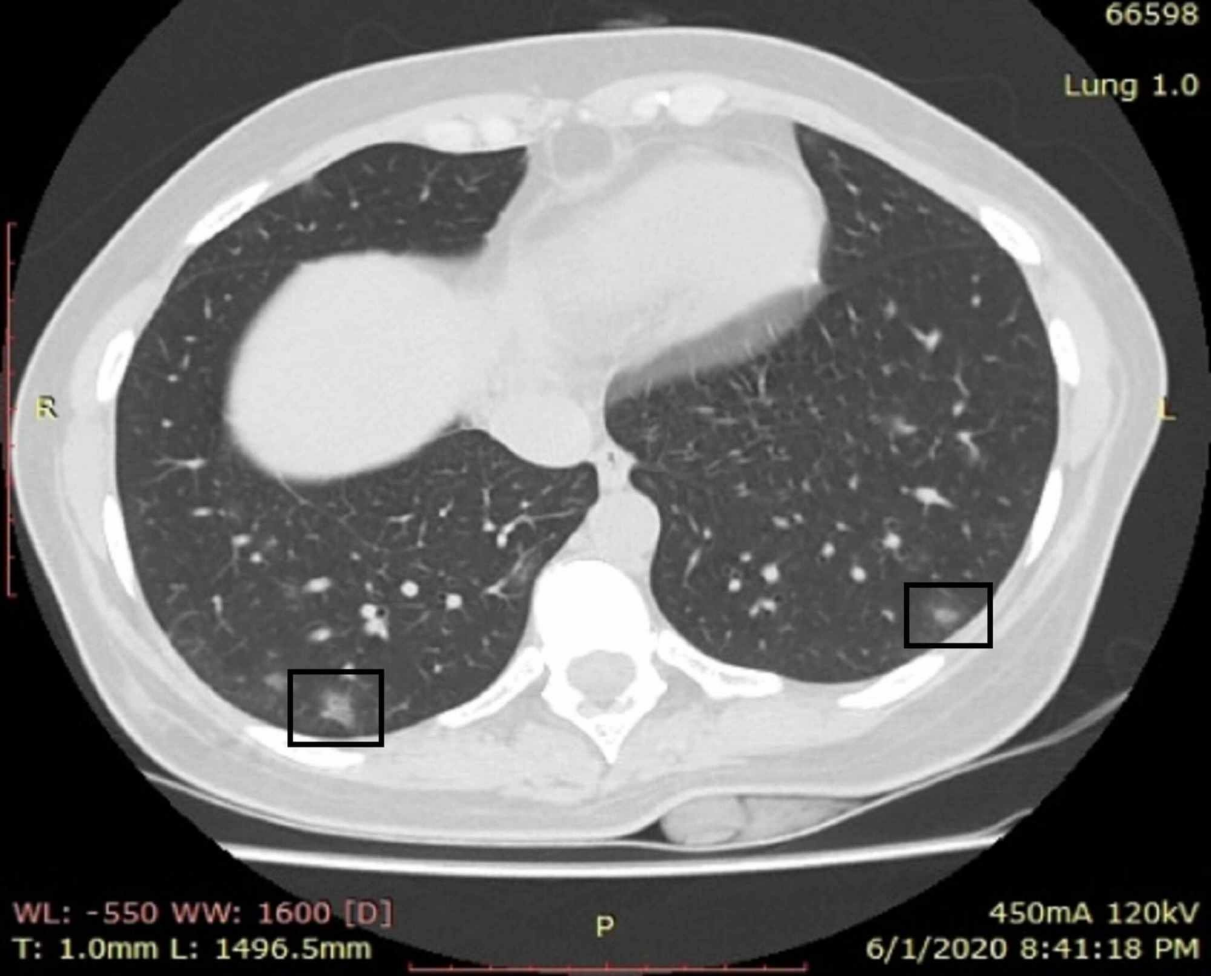
A young female patient with cough and fever who presented on the fifth day of symptoms CT shows small peripheral bilateral areas of ground-glass opacities in lower lobes (boxes). No area of consolidation was identified CT: computed tomography

**Figure 2:**
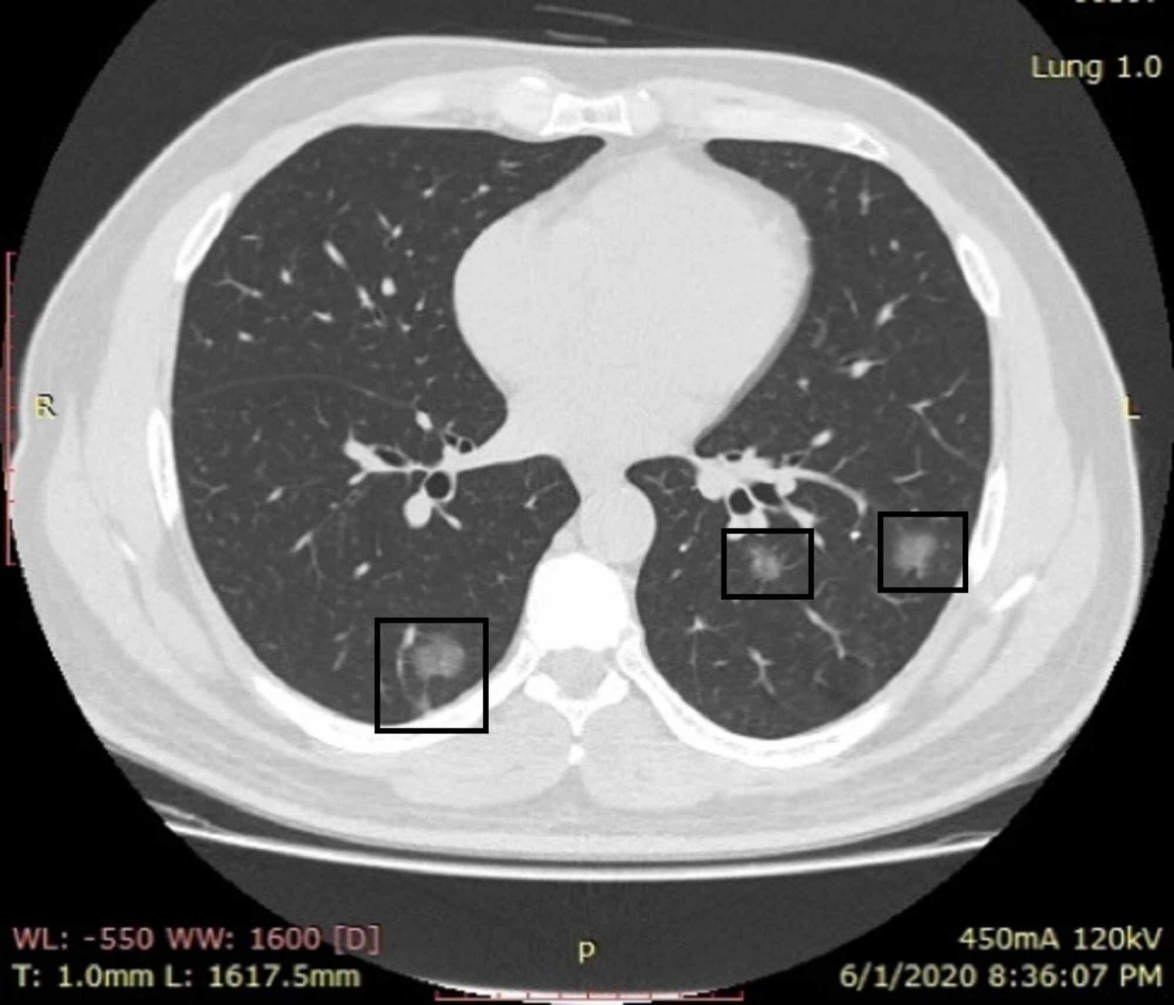
A COVID-19 RT-PCR-positive male patient with apprehensions and no respiratory symptoms CT shows small irregular patches of ground-glass opacities in bilateral lower lung fields (boxes) CT: computed tomography; COVID-19: coronavirus disease 2019; RT-PCR: reverse transcription-polymerase chain reaction

**Figure 3:**
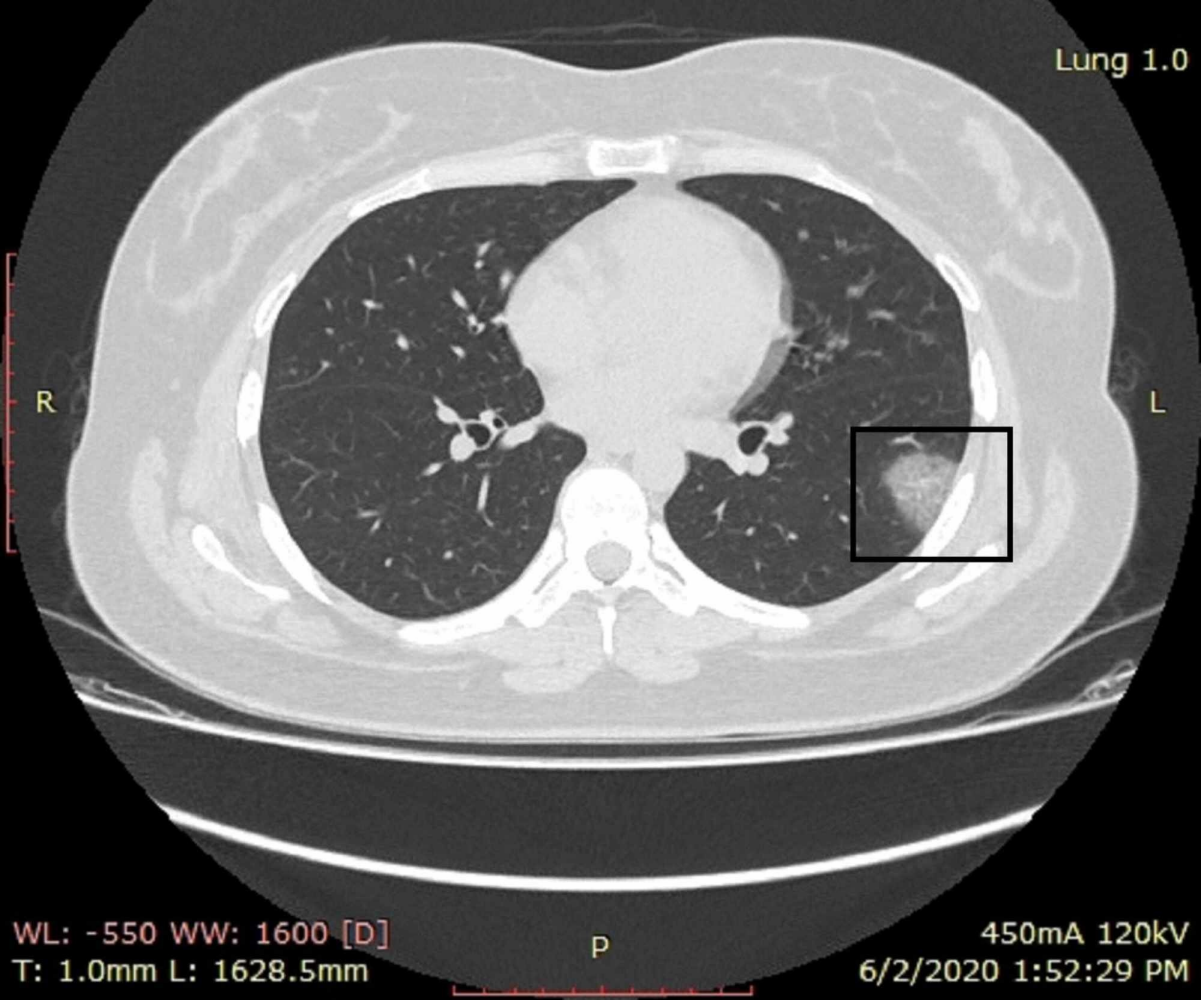
A 30-year-old female patient with mild symptoms CT shows a single patch of ground-glass opacity in the superior segment of the left lower lobe (box) CT: computed tomography

**Figure 4:**
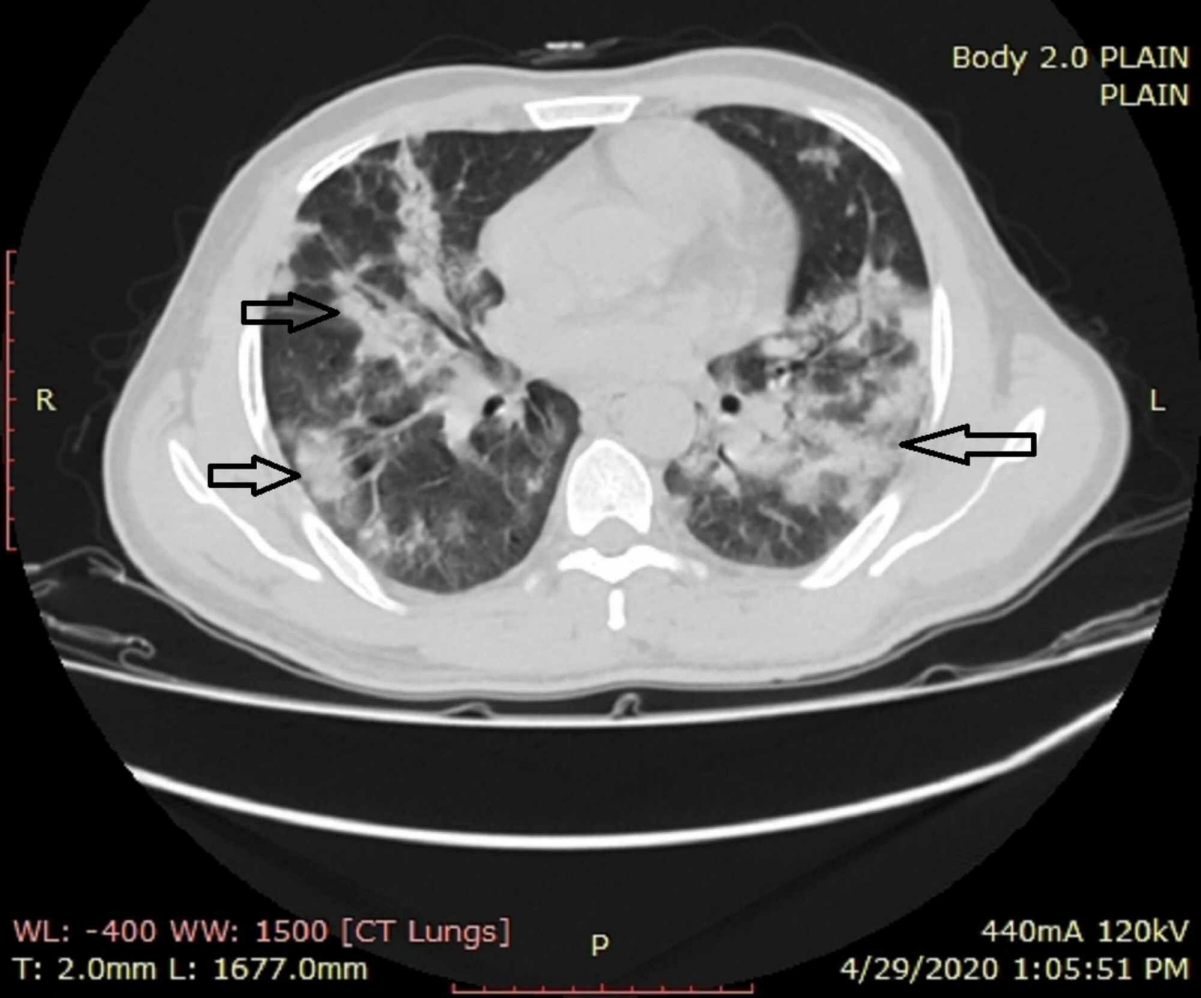
A female patient with dropping oxygen saturation CT shows multiple bilaterally predominant areas of consolidations with interspersed areas of ground-glass opacities in peripheral as well as central distribution (arrows) CT: computed tomography

**Figure 5:**
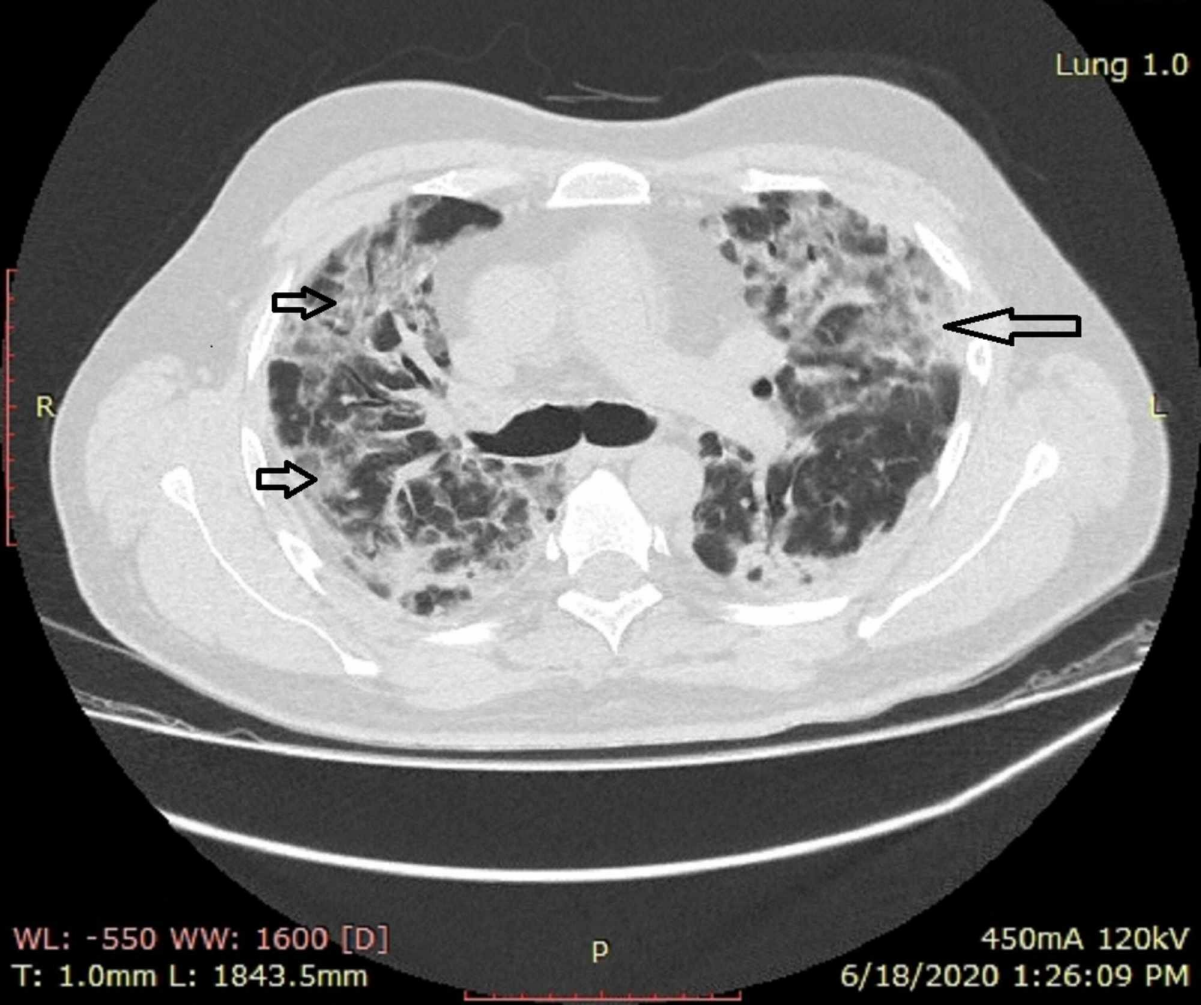
A male patient with severe disease HRCT shows multiple bilateral areas of ground-glass opacities and consolidation in bilateral lungs (short and long arrows) HRCT: high-resolution computed tomography

GGOs were the most common finding, as seen in 88.5% of the patients, followed by consolidations (52.8%) and crazy paving (33.3%) (Figures 1, 2, 3; Table 1). While 37% of patients exhibited a mixed pattern of ground-glass haze and consolidations (Figures 4, 5; Table 1), the majority of the patients showed bilateral and peripheral distribution of the disease process (Figures 1, 2, 3, 4, 5; Table 1). Vascular dilatation and bronchiectasis in diseased parenchyma were seen in 10 study patients. Pleural effusions were noted in only two study patients, while no patient exhibited a reverse halo sign or pulmonary nodules (Table 1).

**Table 1:** Characteristics and manifestations of COVID-19 on high-resolution CT chest GGO: ground-glass opacity; COVID-19: coronavirus disease 2019; CT: computed tomography

Imaging characteristics	Number of patients (% of total)
Ground-glass opacity (GGO)	77 (88.5%)
Consolidation	46 (52.8%)
Mixed pattern (GGO + consolidation)	37 (42.5%)
Crazy paving	29 (33.3%)
Reverse halo sign	0 (0%)
Nodules	0 (0%)
Pleural effusion	2 (2.2%)
Other findings (bronchiectasis and vascular dilatation in diseased parenchyma)	10 (8.7%)
Distribution	
Unilateral	21 (24.1%)
Bilateral	66 (75.8%)
Central	26 (29.8%)
Peripheral	71 (81.6%)
Mixed (central + peripheral)	24 (27.5%)

**Figure 6:**
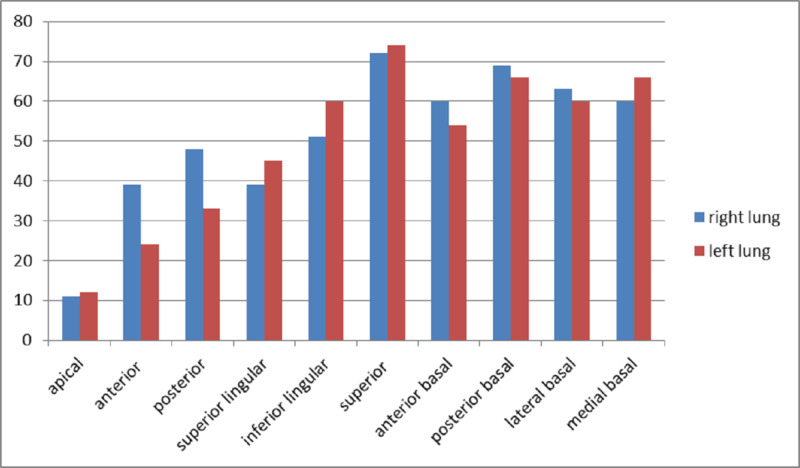
Total CT severity score for each lung segment CT: computed tomography

Superior segment of the lower lobes was the most commonly involved segment bilaterally with involvement noted in 66 patients in the right lung and 69 patients in the left lung (Table 2; Figure 6). The total CT-SS of these segments was 72 and 75 respectively (Table 2). The second-most commonly involved segment was the posterior basal segment in both lungs (Figure 6).

The total CT-SS for the right lung was 512, whereas it was 495 for the left lung (Table 2). According to CT-SS, 78 (89.6%) patients had mild disease having the involvement of less than 50% of lung parenchyma. Six (6.9%) and three (3.45%) patients had moderate and severe diseases, respectively (Table 3).

**Table 2:** Scores for each lung segment of the right and left lung

Right lung zone/score	Number of patients	Total score	Left lung zone/score	Number of patients	Total score
Apical			Apical		
0	76	11	0	75	12
1	11		1	12	
2	0		2	0	
Anterior			Anterior		24
0	51	39	0	66	
1	33		1	18	
2	3		2	3	
Posterior			Posterior		
0	45	48	0	57	33
1	36		1	27	
2	6		2	3	
Medial			Superior lingular		
0	54	39	0	45	45
1	27		1	39	
2	6		2	3	
Lateral			Inferior lingular		
0	45	51	0	30	60
1	33		1	54	
2	9		2	3	
Superior			Superior		
0	21	72	0	18	75
1	60		1	63	
2	6		2	6	
Lateral basal			Lateral basal		
0	30	63	0	33	60
1	51		1	48	
2	6		2	6	
Anterior basal			Anterior basal		
0	33	60	0	39	54
1	48		1	42	
2	6		2	6	
Posterior basal			Posterior basal		
0	27	69	0	27	66
1	51		1	54	
2	9		2	6	
Medial basal			Medial basal		
0	30	60	0	27	66
1	54		1	54	
2	3		2	6	
Total score for right lung	512		Total score for left lung	495	

**Table 3:** Disease severity based on total CT severity score CT: computed tomography

Disease severity	Number of patients	%
Mild disease lung score: 0-20; <50% lung parenchyma	78	89.60%
Moderate disease lung score: 20-30; 50-75% lung parenchyma	6	6.90%
Severe disease lung score: >30; >75% lung parenchyma	3	3.45%

## Discussion

Since the outbreak of coronavirus in December 2019, the population affected by the virus has been on the rise. The affected population may be asymptomatic in many cases. Among symptomatic patients, the spectrum of the disease ranges from mild presentations to serious conditions such as severe acute respiratory syndrome (SARS) [7,9,10]. This disease burden and the consequent threat to public health are well-recognized by the World Health Organization (WHO) [11]. Multisystem involvement has been observed in COVID-19-positive patients with the respiratory system being the most common area to get affected [12]. Respiratory symptoms in the affected patients range from dry cough to respiratory distress [7,13,14]. It is believed that damage to the alveolar epithelium is mainly responsible for acute respiratory distress syndrome (ARDS) seen in COVID-19, while the endothelium of blood vessels is less damaged, thereby resulting in a lesser amount of exudation. This explains the lesser involvement of other organ functions in COVID-19 patients. The diagnostic hallmark of the disease is the reverse transcription-polymerase chain reaction (RT-PCR) test. It has been proven by multiple studies that the test has an overall sensitivity of 61-70% [2,3,4]. However, the test's sampling technique, longer waiting time, and lack of widespread availability have presented some challenges and limitations relating to its use for the prompt and efficient diagnosis of COVID-19. Hence, the CT scan of the chest can play a pivotal role in the diagnosis and management of patients with COVID-19 pneumonia.

The present study showed that the predominant pulmonary parenchymal pattern for this disease is ground-glass haze/opacity followed by consolidation, as seen in 88.5 and 52.8% of patients respectively. This is comparable to the other studies in the Chinese population [15-19]. A study by Chung et al. showed that ground-glass opacification was the most frequently encountered imaging feature, as observed in 86% of the study population. The mixed pattern of both GGOs and consolidations was detected in 29% of the population, compared to 42.5% of the population in our study [5]. The crazy paving pattern was noted in 33.3% of our study population, which was comparable to a study by Kanne et al., which showed crazy paving in 19% of the population [4].

The reversed halo sign is defined as an area of central GGO with a surrounding rim of consolidation [20,21]. The reversed halo sign is seen in many conditions including cryptogenic organizing pneumonia, fungal infections, tuberculosis, sarcoidosis, and many other ailments [22]. A study by Zhao et al. has shown that the CT chest of 25.2% of COVID-19 patients exhibited these signs. Moreover, according to that study, patients with moderate disease exhibited reversed halo sign more than those with severe disease (31.3 vs 13% respectively) [18]. Another study by Jing et al. showed a reversed halo sign in 4.6% of the patients [17]. The findings of our study contrast with these results as they showed no reversed halo sign in any of the patients. This could be attributed to the fact that our study included a limited number of patients with moderate and severe disease.

Bilateral disease was seen in 75.8% of patients, which is also comparable to other studies [4,5,19]. It was observed that patients having unilateral disease (24.1%) had mild disease and had mild or no symptoms, with the onset duration of symptoms never exceeding more than one week. Similarly, the peripheral disease was seen in the majority of the patients (81.65%). The mixed pattern of both peripheral and central disease was exhibited by 27.5% of the patients, and a majority of these patients had either moderate or severe disease, and all of them had some respiratory symptoms.

The scores noted for right and left lung in our study were also comparable with other studies [23]. A study by Yang et al. showed that the most commonly involved segments included posterior basal and superior segments of the lower lobes and posterior segments of the upper lobe [23]. Similar results were shown in our study where the most common segment involved was the superior segment of lower lobes, followed by posterior basal and lateral basal segments of the lower lobe.

There are a few limitations to this study. Firstly, the sample size was small, accounting for a small proportion of the total number of positive cases in Pakistan. Secondly, limited clinical and lab data were available due to the hectic workload in hospitals during this outbreak. Thirdly, the course of the disease and outcomes in participants were not taken into account.

## Conclusions

Based on our findings, the typical imaging findings of COVID-19 pneumonia on HRCT are GGOs with multilobe involvement and bilateral, peripheral, and basal predominance. CT-SS is helpful in categorizing pneumonia into mild, moderate, and severe types, thereby enabling to identify patients with severe disease. This is particularly helpful in settings where fast triage is required.

## References

[1] Li G, Fan Y, Lai Y (2020). Coronavirus infections and immune responses. J Med Virol.

[2] Ai T, Yang Z, Hou H (2020). Correlation of chest CT and RT-PCR testing in coronavirus disease 2019 (COVID-19) in China; a report of 1014 cases. Radiology.

[3] Fang Y, Zhang H, Xie J, Lin M, Ying L, Pang P, Ji W (2020). Sensitivity of chest CT for COVID-19: comparison to RT-PCR. Radiology.

[4] Kanne JP (2020). Chest CT findings in 2019 novel coronavirus (2019-nCoV) infections from Wuhan, China: key points for the radiologist. Radiology.

[5] Chung M, Bernheim A, Mei X (2020). CT imaging features of 2019 novel coronavirus (2019-nCoV). Radiology.

[6] Zhou S, Wang Y, Zhu T, Xia L (2020). CT features of coronavirus disease 2019 (COVID-19) pneumonia in 62 patients in Wuhan, China. Am J Roentgenol.

[7] Huang C, Wang Y, Li X (2020). Clinical features of patients infected with 2019 novel coronavirus in Wuhan, China. Lancet.

[8] Lei J, Li J, Li X, Qi X (2020). CT imaging of the 2019 novel coronavirus (2019-nCoV) pneumonia. Radiology.

[9] Lu R, Zhao X, Li J (2020). Genomic characterization and epidemiology of 2019 novel coronavirus: implications for virus origins and receptor binding. Lancet.

[10] Zhu N, Zhang D, Wang W (2020). A novel coronavirus from patients with pneumonia in China, 2019. N Engl J Med.

[11] (2020). World Health Organization: coronavirus disease (COVID-19) outbreak. https://www.who.int/emergencies/diseases/novel-coronavirus-2019.

[12] Chan JF, Yuan S, Kok KH (2020). A familial cluster of pneumonia associated with the 2019 novel coronavirus indicating person-to-person transmission: a study of a family cluster. Lancet.

[13] Wang D, Hu B, Hu C (2020). Clinical characteristics of 138 hospitalized patients with 2019 novel coronavirus-infected pneumonia in Wuhan, China. JAMA.

[14] Guan WJ, Ni ZY, Hu Y (2020). Clinical characteristics of coronavirus disease 2019 in China. N Engl J Med.

[15] Song F, Shi N, Shan F (2020). Emerging 2019 novel coronavirus (2019-nCoV) pneumonia. Radiology.

[16] Li Y, Xia L (2020). Coronavirus disease 2019 (COVID-19): role of chest CT in diagnosis and management. AJR Am J Roentgenol.

[17] Wu J, Pan J, Teng D, Xu X, Feng J, Chen YC (2020). Interpretation of CT signs of 2019 novel coronavirus (COVID-19) pneumonia (Epub ahead of print). Eur Radiol.

[18] Zhao H, Liang T, Wu CC (2020). The reversed halo sign in COVID-19 pneumonia (PREPRINT). Res Square.

[19] Bernheim A, Mei X, Huang M (2020). Chest CT findings in coronavirus disease-19 (COVID-19): relationship to duration of infection. Radiology.

[20] Hansell DM, Bankier AA, MacMahon H, McLoud TC, Müller NL, Remy J (2008). Fleischner Society: glossary of terms for thoracic imaging. Radiology.

[21] Zompatori M, Poletti V, Battista G, Diegoli M (1999). Bronchiolitis obliterans with organizing pneumonia (BOOP), presenting as a ring-shaped opacity at HRCT (the atoll sign). A case report. Radiol Med.

[22] Godoy MC, Viswanathan C, Marchiori E, Truong MT, Benveniste MF, Rossi S, Marom EM (2012). The reversed halo sign: update and differential diagnosis. Br J Radiol.

[23] Yang R, Li X, Liu H (2020). Chest CT severity score: an imaging tool for assessing severe COVID-19. Radiol Cardiothorac Imaging.

